# miR-218-5p in endometrial microenvironment prevents the migration of ectopic endometrial stromal cells by inhibiting LASP1

**DOI:** 10.1186/s12958-022-00928-z

**Published:** 2022-04-04

**Authors:** Ziyu Zhang, Yaoqing Wang, Liqin Zeng, Kaihui Yu, Yuanqin Wang, Yong Luo, Faying Liu, Bicheng Yang, Yang Zou, Liqun Wang, Ouping Huang

**Affiliations:** 1 Department of Pathology, Jiangxi Maternal & Child Health Hospital, Nanchang, Jiangxi 330006 PR China; 2 Central Laboratory, Jiangxi Maternal and Child Health Hospital, Nanchang, 330006 Jiangxi China; 3 Department of Reproductive Health, Jiangxi Maternal & Child Health Hospital, Nanchang, Jiangxi 330006 PR China; 4 The College of Medicine, Nanchang University, Nanchang, Jiangxi 330006 PR China; 5 Jiangxi Provincial Key Laboratory of Birth Defect for Prevention and Control, Jiangxi Maternal & Child Health Hospital, Nanchang, Jiangxi 330006 PR China

**Keywords:** miR-218-5p, LASP1, Endometriosis, Adenomyosis, Migration

## Abstract

**Background:**

Our previous two-dimensional electrophoresis experiment showed that the expression of LASP1 in patients with endometriosis was significantly higher than that of control endometrium. However, the molecular mechanism by which LASP1 is regulated in endometriosis/adenomyosis is unknown.

**Methods:**

Herein, qPCR was performed to analyze the expression levels of LASP1 and miR-218-5p between endometriosis (Ems) cells and control cells. Fluorescence in situ hybridization was carried out to measure the expression level of miR-218-5p in ectopic endometrium versus normal endometrium. After miR-218-5p mimic or inhibitor were transfected, the transwell experiment was carried out to see the effect of miR-218-5p on the migration of endometrial stromal cells (ESCs). EdU was used to measure cell proliferation rate. Dual-luciferase reporter assay was used to verify the binding of hsa-miR-218-5p to the 3’UTR of LASP1. Western blot and immunofluorescence analysis were carried out to identify the protein expression pattern of LASP1 and EMT markers in endometrial tissue.

**Results:**

The miR-218-5p is mainly secreted from blood vessels and expressed in the muscle layer around the endometrium, which inhibits the expression level of LASP1 by binding the 3’UTR region of LASP1 in normal ESCs. Overexpression of miR-218-5p impedes the epithelial-to-mesenchymal transition (EMT) and prevents the migration of ESCs and the expression of Vimentin in Ems.

**Conclusions:**

Our findings revealed that miR-218-5p in endometrial microenvironment prevents the migration of ectopic endometrial stromal cells by inhibiting LASP1.

**Supplementary Information:**

The online version contains supplementary material available at 10.1186/s12958-022-00928-z.

## Introduction

Although endometriosis/adenomyosis (Ads) are benign diseases, most studies have found that the infiltration and metastasis characteristics of Ems are similar to malignant tumors. Studies have found that the ability of metastatic bladder cancer cell lines to infiltrate and metastasize is similar to that of endometrial cells entering the myometrium [3]. The key point of the infiltration and metastasis of cells is the migration and movement of cells, and the first step of migration is the remodeling of the filamentous actin cytoskeleton, which can promote the formation and adhesion of synapses [23].

Actin backbone protein LIM and SH3 protein1 (LASP1) is a special actin binding and plaque binding scaffold protein, which participates in the migration of cells and plays a critical role in actin assembly, such as adhesion spots, pseudopods and fragmented feet concentrated. The LIM domain at the amino terminus of the LASP1 protein is a zinc finger domain, which is mainly found in transcription factors, signal transduction proteins, and cytoskeleton proteins. As a regulatory binding domain, the LIM domain can mediate between protein–protein interaction. There are two nebulin-like repeats after the zinc finger structure region of LASP1 protein, named R1 and R2 region respectively. As the actin binding region of LASP1, R2 mediates the interaction between LASP1 and the actin at the protrusions of the cell membrane [10, 12, 13, 18], thereby stimulating the movement of cells and promoting cell migration. LASP1 was first found in breast cancer, and was confirmed to be related to the progression and metastasis of breast cancer [19]. Other studies have confirmed that LASP1 is highly expressed in a variety of malignant tumors such as ovarian cancer, liver cancer, colorectal cancer, and pancreatic cancer, and is closely related to the occurrence, development, invasion and metastasis of tumors, and is regarded as an oncogene [7, 21, 22, 29].

MicroRNA (miRNA), which is about 20-25nt long non-coding RNA [4], is a single-stranded non-coding small RNA and has post-transcriptional regulatory activity [1]. Studies have found that LASP1, as a miRNA target gene, is involved in the pathophysiological process of multiple tumors [5]. For example, miR-203 acts on LASP1 to affect the proliferation, invasion, and metastasis of esophageal squamous cell carcinoma, prostate cancer, and human triple-negative breast cancer cells [14, 16, 17, 20]. miR-1, miR-133a, miR-218-5p target down-regulation of LASP1 to inhibit the survival rate of bladder cancer cells [2].

Our primary data suggested that LASP1 in ectopic endometrial tissue of Ems patients was significantly up-regulated, which strongly suggests that LASP1 protein plays an important role in the development of Ems [9]. At present, there is no relevant study on whether miR-218-5p plays a role in Ems. Based on this, for the first time, we plan to use the fluorescence in situ hybridization to further analyze the location of miR-218 in Ems tissue, and explore its mechanism and function in Ems.

## Materials and methods

### Human endometrial stromal cells (ESCs)

All experimental tissue specimens were obtained from patients treated in Jiangxi Maternal and Child Health Hospital (January 2018-January 2019). The control ESCs (ThESC/Group1) was obtained from ATCC (CRL-4003, a hTERT-immortalized endometrium stromal cell line from an adult female with non-malignant myomas). We collected eutopic endometrium from 7 patients (average age, 33.37 ± 3.28) diagnosed with Ems (Endometrial glands and stroma are attached to the ovarian cyst wall), and then ESCs were extracted as the eutopic endometrium group (Q-Z). Successfully extracted and stably cultured Q-Z1 as the Ems eutopic endometrium group (Group2). Similarly, extracted and stably cultured Q-Y19 as the Ems ectopic group (Group3). 3 cases of endometrium from patients (average age, 35.27 ± 3.74) were pathologically diagnosed as Ads (Endometrial stroma and glands invade the myometrium more than 2 mm) and then ectopic endometrium were extracted (X–Y). The X-Y19 was successfully isolated and stably cultured as Ads ectopic endometrium group (Group4).

Endometrial tissue from patients without hormone treatment for > 3 months undergoing hysterectomy from the Jiangxi Maternal and Child Health Hospital (Nanchang, Jiangxi Province, China). The samples have been shown to be in the proliferative phase of the menstrual cycle in pathology and histology. The patients signed a written informed consent form prior to recruitment. This study is in line with the Helsinki Declaration and approved by the Ethics Review Body Committee of the Jiangxi Maternal and Child Health Hospital. Separation, characterization and culture of ESCs were performed as described previously [8].

### Isolation, culture and identification of ESCs

The tissues were cut into pieces and cultured in 10% FBS + DMEM/F12 containing 1 mg/ml type IV collagenase for 1.5 h at 37 °C. After the tissue was digested and filtrated with a cell strainer, the tissue was centrifuged at 1000 rpm for 5 min. The pellet was resuspended and cultured with 10% FBS + DMEM/F12 supplemented with 100 IU/ml penicillin, 50 mg/ml streptomycin. In Supplementary data, cell characterization was assessed by immunofluorescence using anti-Vimentin (1:1000 dilution, Proteintech Group) and anti-E-cadherin (1:1000 dilution, BD Transduction Laboratories) antibodies.

### Plasmids and miRNA

The mimic and inhibitor of miR-218-5p were synthesized by Ribo Biotechnology (Guangzhou, China). The dual-luciferase reporter plasmid of LASP1 3’UTR widetype or mutant sequence (207 bp) was subcloned into GV272 vector with the XbaI/XbaI restriction endonuclease by Genechem (Shanghai, China).

### Western blot

Western blotting was performed as previously described [27, 28]. To measure the endogenous protein level, ESCs were transfected with control mimic/inhibitor or miR-218-5p mimic/inhibitor, respectively. After 48 h, RIPA buffer (50 mM Tris–HCl, pH 7.5, 150 mM 475 M NaCl, 1% Nonidet P-40, 0.5% sodium deoxycholate, 0.1% SDS, 1xRoche complete protease inhibitor mixture) was used to lysis cell. Protein (about 20–30 μg/line) was separated by SDS-PAGE gel and transferred onto a PVDF membrane (Millipore, CA, USA), then blocked with 5% milk powder, and incubated with anti-LASP1 antibody (1:1000 dilution, rabbit, ab191022, Abcam) and anti-Vimentin (1:2000 dilution, Cat No: 10366–1-AP, Proteintech) and overnight at 4 °C. The membrane was washed three times with 1xTBS-T and incubated with horseradish peroxidase (1:5000) secondary antibody for 1 h at room temperature. Protein bands were visualized by ECL system (Thermo Fisher Scientific, IL, USA). The protein expression levels were normalized to the expression of GAPDH (1:2000 dilution, Cat No: sc-47724, Santa Cruz Biotechnology) in each line.

### Cell migration assay

Cell migration assay was carried out as described previously [27, 28]. For assessing ESCs cell migration, 1 × 10^4^ cells in serum free media were seeded into the transwell inserts (Corning) and allowed to migrate toward 10% FBS-containing medium. Later, the cells in the transwell inserts were removed, and the inserts were washed in 1xPBS three times. The migrated cells on the bottom of the insert were fixed with methanol solution followed by crystal violet (1%) staining. After washing the inserts 3 times with 1xPBS, the inserts were allowed to air dry and pictures were taken by using Olympus X71. Six independent fields were counted for each transwell and the average number of penetrating cells was represented in the graphs.

### EdU incorporation assay

ESCs cells were seeded onto 24 well plate after transfected with miRNA mimic or inhibitor. Next day, cells were incubated with 10 μM Edu for 6 h, washed 3 times wtih 1xPBS, fixed in 3.7% formaldehyde for 15 min at room temperature, and followed by Edu insertion detection according to the manufacturer instructions (Guangzhou RiboBio Co., Ltd, China) [6].

### Immunofluorescence

The paraffin-embedded adenomyosis tissue sections were baked in the oven at 65 °C for 12 h. After deparaffinization and blocking, the antigen–antibody reaction was incubated overnight at 4 °C. Immunofluorescence staining with following antibodies: anti-Vimentin (Cat No: 10366–1-AP, Proteintech), anti-E-cadherin (Cat No: 610181, BD, Biosciences), anti-VEGF (Cat No: 19003–1-AP, Proteintech), anti-SMA (Cat No: 55135–1-AP, Proteintech). Fluorescence was visualized with Olympus X71.

### RNA isolation and qPCR

We carried out the RNA isolation as described previously [26]. The total RNAs were extracted from ESCs by using the RNAiso reagent (TaKaRa, Shiga, Japan). The reverse transcription reaction was also performed by using the PrimeScript reagent Kit (TaKaRa). Real-time quantification PCR reaction was carried out by using the SYBR Premix Ex TaqII (TaKaRa) on the 7500 Real-Time PCR System (Applied Biosystems) with primers for: RT-LASP1-F’: AACGCACACTACCCCAAG, RT-LASP1-R’: CCACTACGCTGAAACCTTTG. RT-Actin-F’: ACCTTCTACAATGAGCTGCG, RT-Actin-R’: CCTGGATAGC-AACGTACATGG. The miR-218-5p (MQPS0000843) and U6 (MQPS0000002) primers were purchased from Ribo Biotechnology (Guangzhou, China). Experiments were repeated at least three times.

### miRNA fluorescence in situ hybridization

The probe for miR-218-5p was purchased from Boster Bioengineering Co., Ltd. (Wuhan, China), and the sequence is 5’-ACATGGTTAGATCAAGCACAA-3’. After deparaffinization, the slides were incubated in 100 μl of 3% citric acid dilution of pepsin and digested for 15 min at room temperature. The slides were washed with 1xPBS 3 times for 5 min and distilled water for once. 1% paraformaldehyde/PBS was used to fix for 10 min, and the samples were washed 3 times with distilled water. The slides were incubated in 20 ul pre-hybridization buffer in a humidified chamber at 42 °C for 2 h. Then the slides were washed with 2x, 0.5 × and 0.2 × SSC (15 min each wash) at 37 °C. The samples were then incubated for 15 min in a blocking solution followed by incubation for 60 min in biotinylated mouse anti-digoxigenin solution. The samples were washed with 1xPBS for 4 times (5 min each wash). Finally, the slides was incubated in SABC-cy3 solution for 30 min, then washed with 1xPBS for 3 times (5 min each wash).

### Statistical analysis

Statistical analyses were carried out by using SPSS 24.0 software. Data were presented as mean ± SEM. To determine the statistical difference of target genes mRNA expression between control group and Ems group by using Student’s t-test. All *p*-values were two-tailed, and *p* < 0.05 were considered to statistical significance(*).

## Results

### miR-218-5p inhibits LASP1 in ESCs

**Fig. 1:**
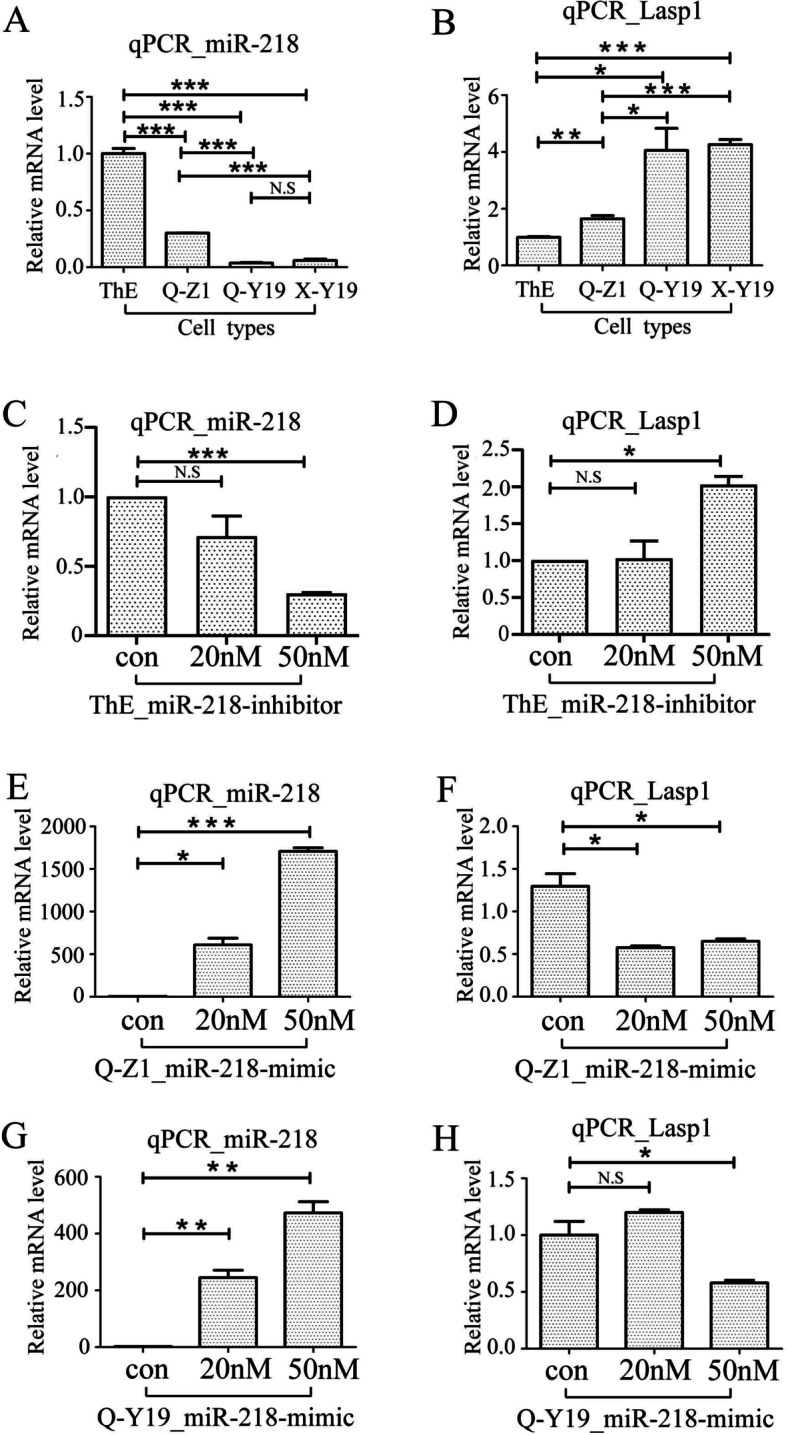
miR-218-5p inhibits LASP1 mRNA level in ESCs. **A** and **B** qPCR analysis of miR-218-5p and LASP1 mRNA levels in different ESCs, respectively. ThE: Eutopic stromal cell line of the control endometrium. Q-Z1: Primary eutopic stromal cell of the endometriosis endometrium. Q-Y19: Primary ectopic stromal cell of the endometriosis endometrium. X-Y19: Primary ectopic stromal cell of the adenomyosis endometrium. **C** and **D** qPCR analysis of miR-218-5p and LASP1 mRNA levels in ThESC transfected with the control inhibitor, 20 nM and 50 nM miR-218-5p inhibitor respectively. **E** and **F** qPCR analysis of miR-218-5p and LASP1 mRNA levels in Q-Z1 transfected with the control inhibitor, 20 nM and 50 nM miR-218-5p inhibitor respectively. **G** and **H** qPCR analysis of miR-218-5p and LASP1 mRNA levels in Q-Y19 transfected with the control inhibitor, 20 nM and 50 nM miR-218-5p inhibitor respectively. P-values were determined by Student’s t-test, *p < 0.05, **p < 0.01, ***p < 0.001

First, we used qPCR to detect the expression of miR-218-5p and LASP1 in different ESCs. We found that the expression level of miR-218-5p was the highest in ThESC(ThE) cells, followed by the eutopic stromal cells Q-Z1 of patients with Ems, and the lowest expression in the two ectopic stromal cells Q-Y19 and X-Y19. On the contrary, in Q-Y19 and X-Y19 cells, the expression level of LASP1 was the highest, followed by Q-Z1, and the expression level of miR-218-5p in ThE was the lowest (Fig. 1A and B). There is no difference in the expression of miR218 and LASp1 between Q-Y19 and X-Y19 cells. The above results indicate that miR-218-5p is lowly expressed in the eutopic mesenchymal cells of patients with non-endometriosis, is moderately expressed in the eutopic mesenchymal cells of patients with Ems, and is expressed at a high level in the ectopic mesenchymal cells of patients with Ems. Moreover, the expression pattern of LASP1 is opposite to miR-218-5p, showing a negative correlation.

Next, we transfected the mimics or inhibitors of miR-218-5p to detect its effect on the expression pattern of LASP1 in the cells with low or high concentrations of miR-218-5p, respectively. Only 50 nM (not 20 nM) miR-218-5p inhibitor decreased the level of miR-218-5p while increased the level of LASP1 in ThESCs (ThE) cell (Fig. 1C and D). 50 nM miR-218-5p mimic increased the level of miR-218-5p while decreased the level of LASP1 in both Q-Z1 and Q-Y19 cell (Fig. 1E-H).

**Fig. 2:**
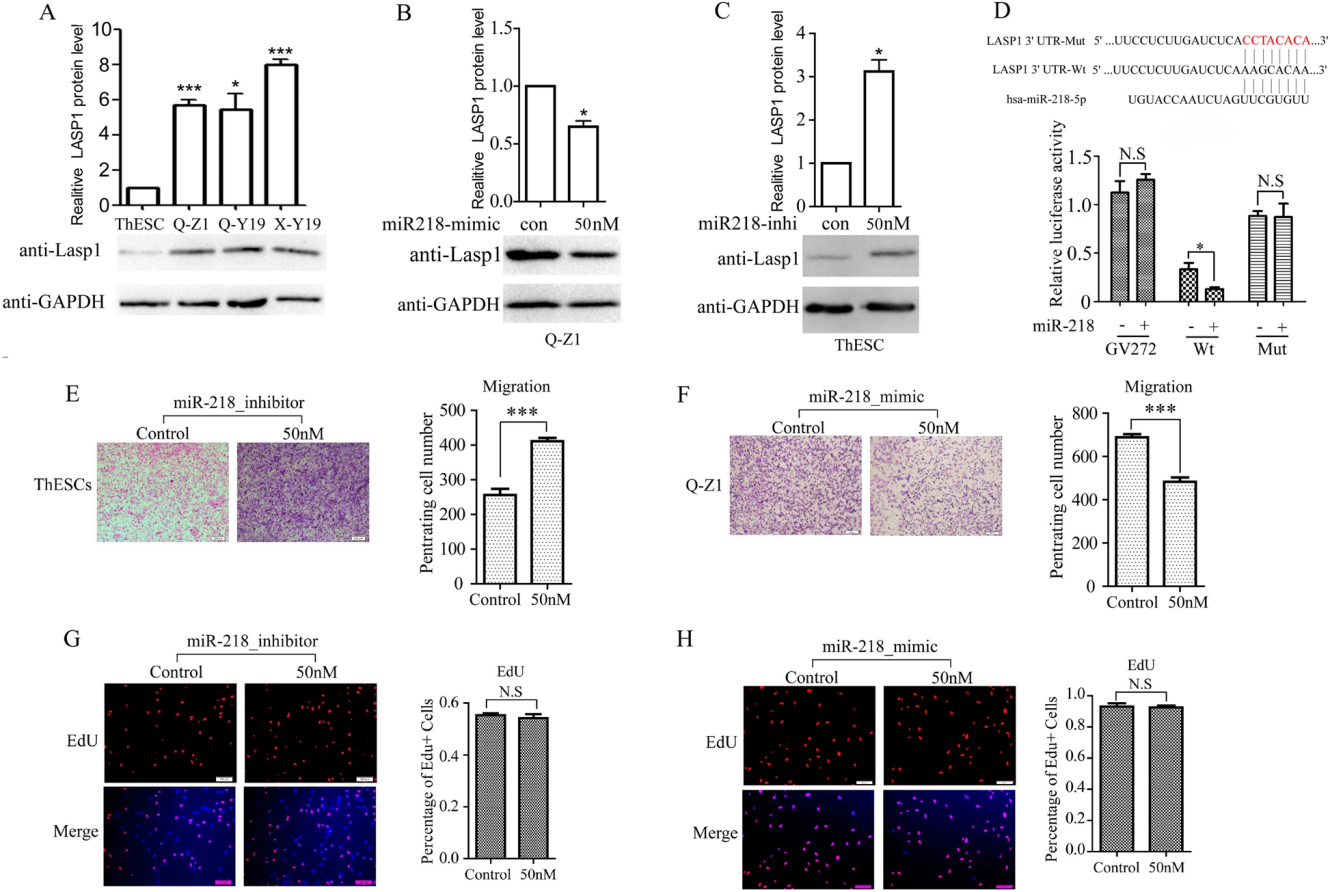
miR-218-5p has no effect on the proliferation of ESCs but can inhibit the migration of ESCs. **A** Western blot analysis and quantification of LASP1 protein level in lysates of different ESCs. **B** Western blot analysis and quantification of LASP1 protein level in lysates of Q-Z1 with or without 50 nM miR-218-5p mimic. **C** Western blot analysis and quantification of LASP1 protein level in lysates of ThESc with or without 50 nM miR-218-5p inhibitor**.** GAPDH was used as the loading control. *P*-values were determined by Student’s t-test, *p < 0.05, ***p < 0.001. **D** Predicted miR-218-5p target recognition sites in the 3’-UTR of human LASP1. Mutation in the miR-218-5p target site is red. 3’UTR of LASP1 luciferase constructs, as indicated, were transfected into 293 T cells together with indicated plasmid for 48 h and subjected to a luciferase reporter assay. The results were normalized to the Renilla luciferase activity and are expressed as the fold change in relative luciferase activity compared with the control. **E** ThE cells were transfected with control inhibitor or miR-218-5p inhibitor. **F** Q-Z1 cells were transfected with control mimic or miR-218-5p mimic. After 24 h of transfection, cells were starved for 24 h before cell migration assay was performed without matrigel transwell filters, Scale bar = 200 μm. The migrated cells were stained and counted. Quantification was done in and is shown with counting six nonoverlaping fields. **G** and **H** EdU incorporation assays of ThE or Q-Z1 cells were transiently transfected with control inhibitor/miR-218-5p inhibitor or control mimic/miR-218-5p mimic, DAPI staining was included to visualize the cell nucleus (Blue), Scale bar = 100 μm. Each bar indicates mean ± SEM. of a representative experiment performed in triplicate. *P*-values were determined by Student’s t-test. *** *p* < 0.001

Similarly, we used western blot to detect the protein level of LASP1 in different ESCs cells. The highest protein expression level is X-Y19, followed by Q-Z1 and Q-Y19, and ThESC is the lowest (Fig. 2A), which echoes the results of qPCR (Fig. 1B). At the protein level, when miR-218-5p mimic was transfected into Q-Z1 cells (a cell with low expression of miR-218-5p), we found that the expression of LASP1 was decreased (Fig. 2B). On the contrary, when miR-218-5p inhibitor was transfected into ThE cells (a cell with high expression of miR-218-5p), we found that the expression of LASP1 was increased (Fig. 2C). The above results indicate that miR-218-5p indeed inhibit the expression of LASP1 in ESCs.

MicroRNA can inhibit the post-transcriptional activity of the target gene by binding to its 3’UTR. Therefore, we constructed the luciferase reporter gene plasmids of the wild type 3’UTR and the mutant 3’UTR of LASP1 (Fig. 2D). Then, miR-218-5p was co-transfected with the above-mentioned reporter-gene plasmid into 293 T cells for luciferase reporter gene activity assay. The results showed that miR-218-5p can inhibit the activity of the wt plasmid, but fail to change the activity of the mutant plasmid (Fig. 2D).

### miR-218-5p has no effect on the proliferation of ESCs but can inhibit the migration of ESCs

In order to further study the function of miR-218-5p in ESCs, we transfected miR-218-5p inhibitor into ThE cells with high expression of miR-218-5p, and performed transwell assay and Edu insertion experiments to detect the changes of cell migration and proliferation respectively. The results showed that after the inhibitor of miR-218-5p was transfected, the migration of ThE cells increased (Fig. 2E), but the proliferation of ThE cell did not altered (Fig. 2G). On the contrary, we transfected the mimic of miR218 in Q-Z1 cells with low miR-218-5p expression, and also carried out transwell assay and Edu insertion experiment to detect the changes of cell migration and proliferation. The results showed that after the mimic of miR-218-5p was transfected the migration of Q-Z1 cells decreased (Fig. 2F), but proliferation of Q-Z1 cell remained unchanged (Fig. 2H).

### miR-218-5p is mainly expressed in the vascular endothelial cells and muscle layer near the endometrium

**Fig. 3:**
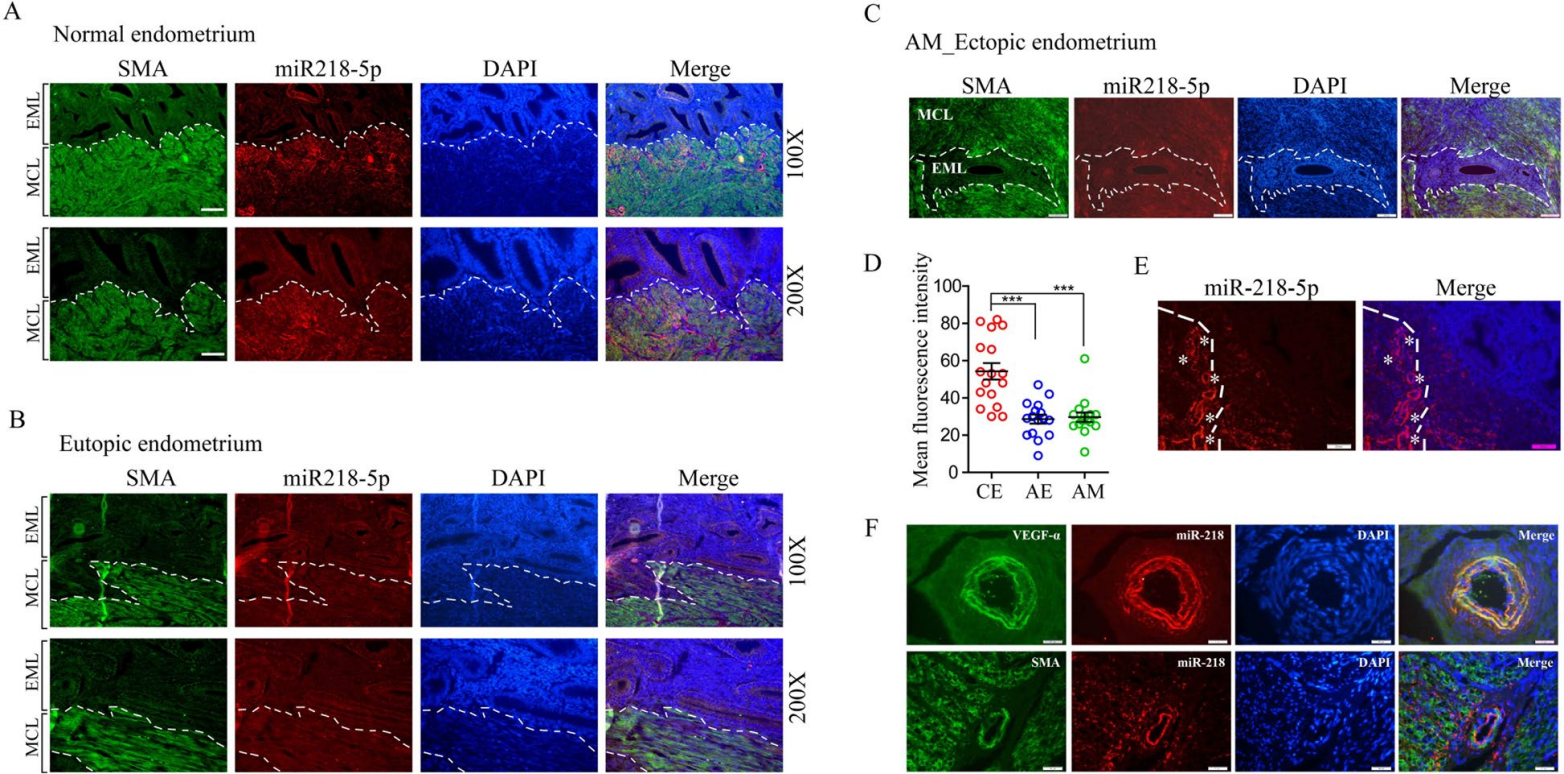
miR-218-5p was overexpressed in myometrium close to the endometrium of CEu. Immunofluorescent analysis of miR-218-5p (Red) and SMA(Green) in the (**A**) control eutopic endometrium (CEu), (**B**) adenomyosis eutopic endometrium(AEu) and (**C**) adenomyosis ectopic endometrium(AEc) respectively. EmL: endometrial layer, *means myometrium, DAPI staining was included to visualize the cell nucleus (Blue), Scale bar = 100 μm. **D** Statistic analysis of Immunofluorescent staining of miR-218-5p in the myometrium of CEu (*n* = 17), AEu (*n* = 16) and AEc (*n* = 16). *P*-values were determined by Student’s t-test, ***p < 0.001. **E** Immunofluorescent analysis of miR-218-5p (Red) in the control endometrium tissue. **F** Immunofluorescent analysis of miR-218-5p (Red), VEGF (Green) and SMA (Green) in the control endometrium tissue. DAPI staining was included to visualize the cell nucleus (Blue), Scale bar = 50 μm

The above experiment show that miR-218-5p can prevent cell migration by inhibiting LASP1 in ESCs. So what is its expression and localization pattern in the endometrial tissue, and is there any difference in expression between the control endometrium and the endometrium of patients with Ems? Next, we performed RNA fluorescence in situ hybridization to detect the expression and localization of miR-218-5p in endometrial tissue (sTable1 and sTable2). In the control eutopic endometrium tissue(CEu), which is very interesting that miR-218-5p is not expressed in endometrial layer (EmL), but mainly expressed in the muscle layer (MCL) close to the endometrium, Show a form of scattered distribution in the cytoplasm (Fig. 3A). However, in adenomyosis eutopic (AEu) and ectopic(AEc) endometrial tissues, miR-218-5p showed low expression in both the endometrium layer and muscular layer (Fig. 3B and C). These results indicate that miR-218-5p is upregulation in the MCL of CEu (Fig. 3D). And we also found that miR-218-5p has strong signal around blood vessels (Fig. 3E). We used VEGF-α and SMA antibodies to label smooth muscle (including vascular smooth muscle) and vascular endothelial, respectively. We found that miR-218-5p mainly co-localized with endothelial cells (Fig. 3F), but not with smooth muscle cells (Fig. 3F). These results suggest that miR-218-5p may be secreted by vascular endothelial cells.

### miR-218-5p can hinder the epithelial-mesenchymal transition

**Fig. 4:**
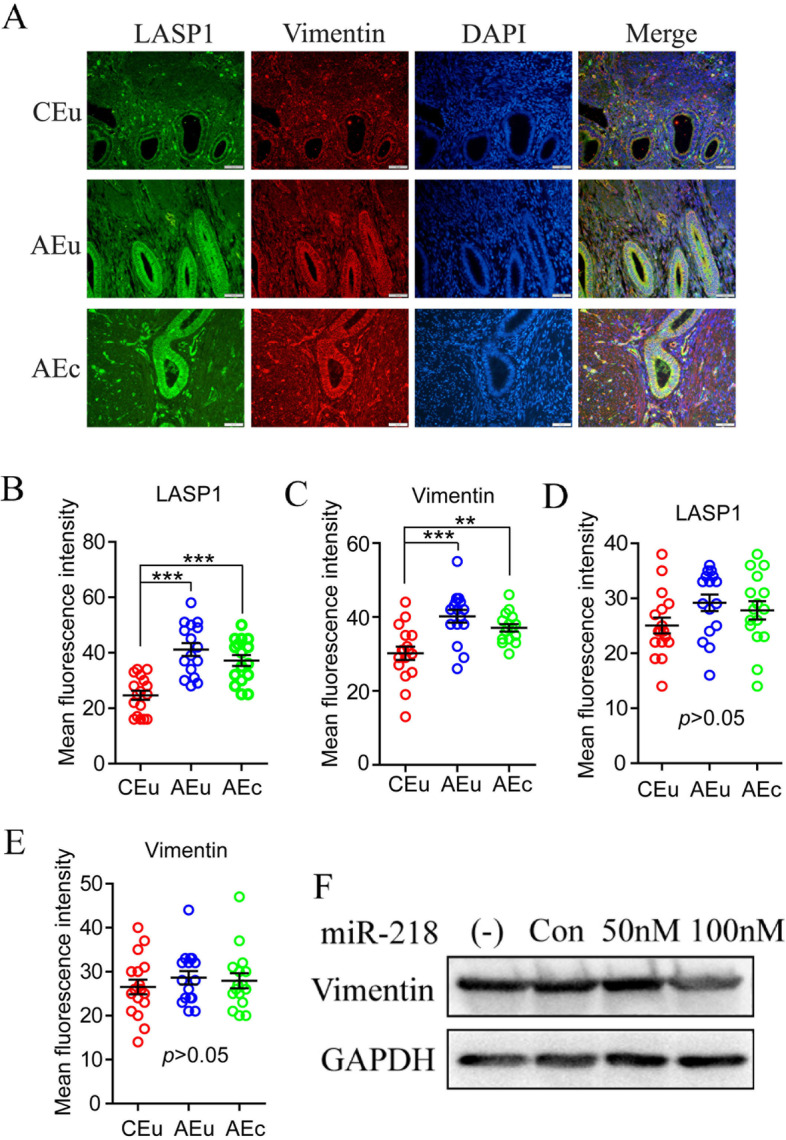
Inhibition of miR-218-5p prevents the epithelial-mesenchymal transition. **A** Immunofluorescent analysis of Vimentin (Red) and LASP1 (Green) in the control eutopic endometrium (CEu), adenomyosis eutopic endometrium(AEu) and adenomyosis ectopic endometrium(AEc) respectively. DAPI staining was included to visualize the cell nucleus (Blue), Scale bar = 100 μm. **B** and **C** Statistic analysis of Immunofluorescent staining of LASP1 and VEGF-α in the gland epithelial cell of three groups. **D** and **E** Statistic analysis of Immunofluorescent staining of LASP1 and VEGF-α in the stromal cell of three groups: CEu (*n* = 17), AEu (*n* = 16) and AEc (*n* = 16). *P*-values were determined by Student’s t-test, ***p < 0.001. **F** Western blot analysis showing effects of miR-218-5p on protein levels of Vimentin. GAPDH was used as the loading control

LASP1 can increase tumor cell migration and invasion by promoting epithelial-mesenchymal transition [22, 30]. We observed the expression and localization of LASP1 and Vimentin (a mesenchymal cell marker) in endometrial tissue. The results showed that the expressions of LASP1 and Vimentin in the eutopic and ectopic endometrial gland epithelial cells of patients with Ems were significantly elevated than those of the control endometrium (Fig. 4A-C). But there is no obvious difference between the three groups in the overall stromal cells, but the scattered and aggregated high expression in the AEc stromal cells (Fig. 4D and E). The above results indicate that the endometrial epithelial cells of Ems already have mesenchymal characteristic. We transfected miR-218-5p mimic into ThE cells to detected the expression of Vimentin by Western blot and found that high concentration of miR-218-5p (100 nM) can significantly inhibit the protein expression level of Vimentin (Fig. 4F). Therefore, miR-218-5p can hinder the EMT by inhibiting Vimentin.

### The protein level of Vimentin was high in adenomyosis epithelial cells

**Fig. 5:**
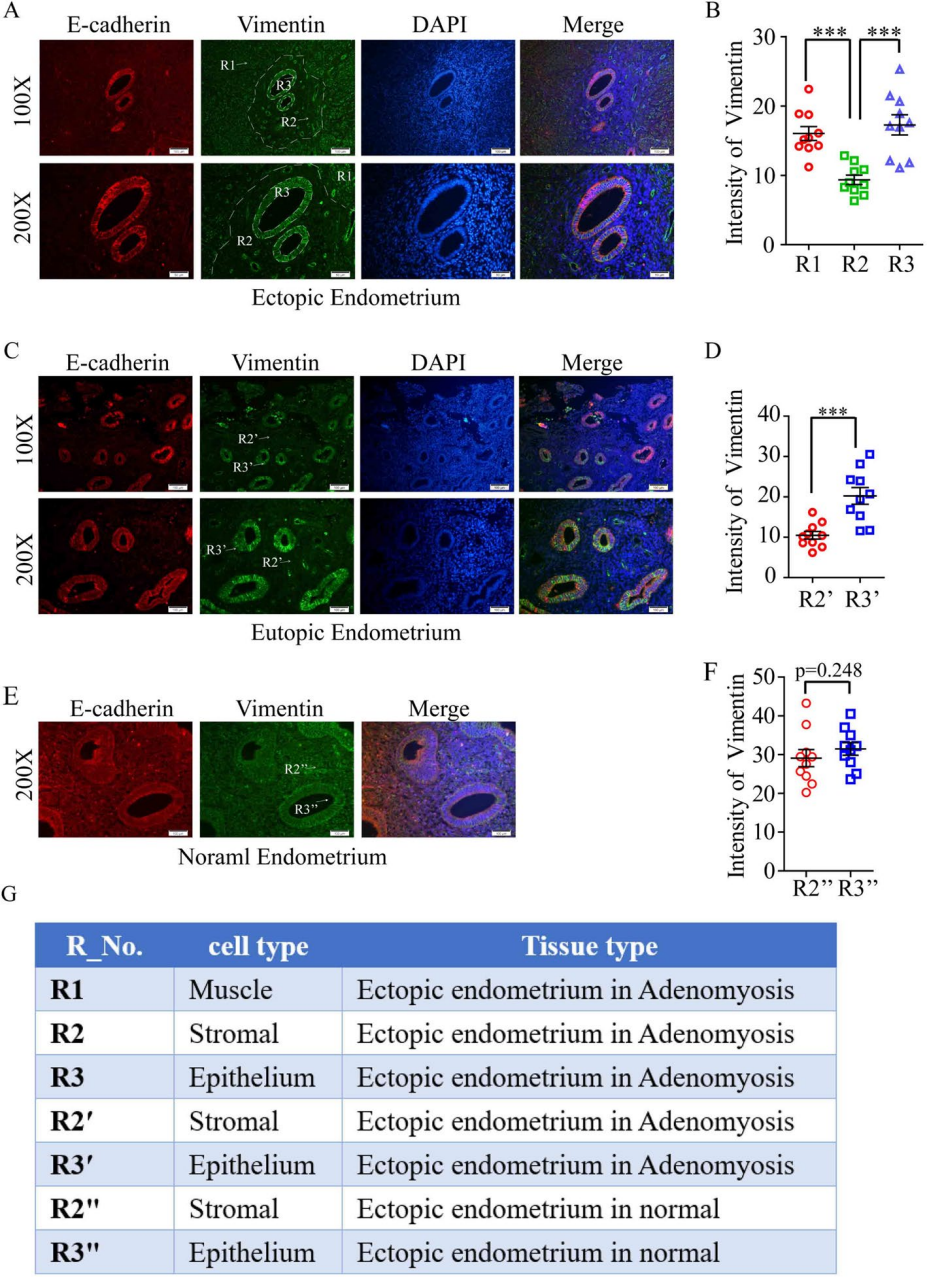
Both E-cadherin and Vimentin were highly expressed in Ems epithelial cells. **A** Immunofluorescent analysis of E-cadherin (Red) and Vimentin (Green) in the ectopic endometrium of Ems tissue. **B** Statistic analysis of Immunofluorescent staining of Vimentin in the region of R1, R2 and R3 Ems tissue(*n* = 10). **C** Immunofluorescent analysis of E-cadherin (Red) and Vimentin (Green) in the eutopic endometrium of Ems tissue. **D** Statistic analysis of Immunofluorescent staining of Vimentin in the region of R2’ and R3’ from Ems tissue (*n* = 10). Scale bar = 100 μm for upper picture and 200 μm for below picture. DAPI was used to stain nuclei. *P*-values were determined by Student’s t-test, ***p < 0.001. **E** Immunofluorescent analysis of E-cadherin(Red) and Vimentin(Green) in the normal endometrium tissue. **F** Statistic analysis of Immunofluorescent staining of Vimentin in the region of R2’’ and R3’’ from normal endometrium tissue(*n* = 10). Scale bar = 50 μm. DAPI was used to stain nuclei. *P*-values were determined by Student’s t-test. **G** A summary of the different R zones

**Fig. 6:**
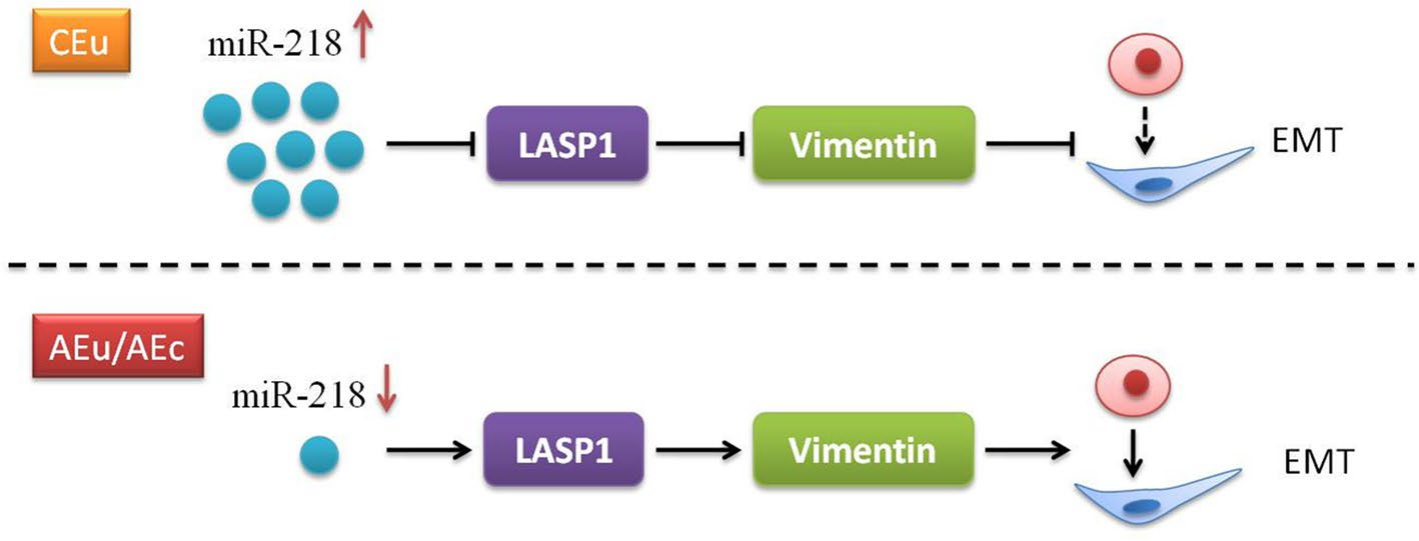
The diagram of miR-218 regulating EMT of endometrial cells. In CEu, the highly expressed miR-218 can inhibit LASP1 in stromalcells, thereby reducing the expression of Vimentin, and ultimately hinder the process of EMT. On the contrary, in AEu/AEc, the low expression of miR-218 promotes the overexpression of LASP1, Which in turn caused the rise of Vimentin and the acceleration of the EMT process

In order to investigate the increase of Vimentin in endometrial gland epithelial cells of Ems, we used Vimentin and E-cadherin (epithelial cell marker) antibodies to perform the immunofluorescence in ectopic and eutopic endometrial tissue sections of adenomyosis patients. In order to verify whether these two antibodies (Vimentin and E-cadherin) are feasible, we tested them for immunohistochemistry in stromal and epithelial cells, and found that E-cadherin is specifically expressed on the membrane of epithelial cells, while Vimentin is specifically expressed in stromal cells (sFig 1), indicating that the expression pattern of these two antibodies is credible. The Fig. 5A and B (Ectopic Endometrial) showed that the expression level of Vimentin in stromal cells (R2) was significantly lower than that in epithelium (R3) and muscle layer (R1). Similarly, in eutopic endometrial tissue sections, the expression level of Vimentin in stromal cells (R2’) was also lower than that in epithelium (R3’) (Fig. 5C and D). Simultaneously, the E-cadherin was only expressed in gland epithelial cells (Fig. 5A and C). The above results indicate that both Vimentin and E-cadherin are highly expressed in the glandular epithelium. In addition, we also found that the expression level of Vimentin in stromal cells of the normal adenomyosis tissue is indistinguishable from that of the epithelial cells (Fig. 5E and F). Taken together, when miR-218-5p was up-regulated, Vimentin in stromal cell were decreased, which was consistent with the expression pattern of Vimentin in tissue sections of CEu. Our results suggested that miR-218-5p inhibitor resulted in adenomyosis epithelial cells transformed into stromal cells through a EMT process. In summary, the abundant miR-218 in blood vessels is secreted into surrounding muscle cells in CEu, and these muscle cells deliver miR-218 to the nearby target tissue–-endometrium, so the expression of miR-218 in stromal cells in CEu was significantly higher than that in AEu/AEc. The highly expressed miR-218 can in turn reduce the expression of Vimentin by inhibiting LASP1 and the reduction of Vimentin as a key protein means the hindrance of EMT, which ultimately inhibits the ability of interstitial cells to migrate excessively (Fig. 6).

## Discussion

As a member of the LIM protein subfamily, LASP1 protein, which interacts with the protruding actin on the cell membrane through the mediation of the LIM structure, can also play a role in the formation, extension and invasion of cell pseudopods through the SH3 region [15]. Relevant studies have confirmed that LASP1 is highly expressed in a variety of malignant tumors, and affects the development, invasion and metastasis of tumors. Although Ems are benign diseases, they have the characteristics of malignant tumor invasion and metastasis. The qPCR results showed that ESCs from ectopic endometrium (whether from adenomyosis or endometriosis) appear to be similar in LASP1 and miR expression (Fig. 1A and B), suggesting that perhaps these are related diseases merely involving different anatomical sites. Based on our preliminary data, we found that the expression level of LASP1 protein was significantly increased in ectopic ESCs of Ems patients. Therefore, it is speculated that LASP1 affects the migration of ESCs and promotes the pathogenesis of Ems. Because Ads and Ems are both endometriotic diseases, this study also compared Ads ESCs (X-Y19) with OEms ESCs (Q-Y19). Many studies have confirmed that LASP1, a downstream target gene of miRNA, which is involved in the process of a variety of malignant tumors. It was found that the expression level of LASP1 was relatively consistent with miR-218 in different ESCs (Fig. 1A and B). The intermediate expression of mR-218-5p in the eutopic stromal cells suggests that such patients may be predisposed to development of endometriosis following reflux of the subset of cells with abnormal gene expression. providing a theoretical basis for the determinism of eutopic endometrium.

Through our qPCR and WB data, it was found that LASP1 in Ads and Ems was highly expressed, while miR-218 was expressed at a low level (Figs. 1A-B and 2A). Besieds, the expression level of miR-218 and LASP1 expression were negative correlation. The results obtained verify our conjecture that LASP1 is highly expressed in Ems, and the mechanism is that miR-218 binds to the 3′UTR region of LASP1 and negatively targets and regulates LASP1, which are consistent with the results of dual luciferase reporter gene assay. And in this case, low expression of miR-218 enhanced the migration of ESCs. This data is consistent with the results of other studies on the function of LASP1 on cell migration. Studies also show that miR-218 exhibits an inhibitory effect on some cervical cancer cell lines (CaSki, HeLa and Yumoto, but not ME180) by reducing cell migration and invasion rather than proliferation [24]. Considering that although Ems has the characteristics of malignant tumor, it is still a benign disease and may not has the characteristics of strong continuous proliferation like malignant tumor.

Based on the expression pattern of LASP1 and miR-218 in different endometrial stromal cells in this study, abnormal gene expression in stromal cells of patients with Ems can be analyzed. This can also explain why some menstrual blood reflux did not cause Ems. The main reason is that these kind of endometrium with menstrual blood reflux has no special biological functions compared with normal endometrium and only the cells with gene expressions are abnormal, even with strong migration and invasion ability can be planted on the basis of menstrual blood reflux, which leads to the pathogenesis of Ems.

For the detection of tissue immunofluorescence, it is interesting that the differential expression of miR-218 does not appear in the endometrium (both stromal and epithelial cells). However, a strong immunofluorescence signal was obviously found around the endometrium (Fig. 3), indicating that miR-218 may be transferred to the microenvironment through exosomes secreted from blood vessels, and further act on the endometrium, changing the physiological state of the endometrium [11, 25]. This transmission signal is a paracrine signal from secretory cell to the target cell, therefore, miR-218 is hard to detect in target cell (endometrium cell). It may not be differentially expressed in the endometrium by immunofluorescence experiment, but in our qPCR results (Fig. 1). Besides, based on the high expression pattern of miR-218 in Ems and Ads stromal cells in our study, indicating that the biological activity of stroma cells has been changed. From the results of the fluorescence in situ hybridization experiment, it can be seen that miR-218 expressed in the muscle layer around the endometrium are the stromal cells, indicating that the stroma cells rather than glands may be the key regulator of endometrial invasiveness and other properties.

Moreover, we also found that miR-218 play a role in EMT. Then, in ectopic endometrial tissue, both E-cadherin and Vimentin were significantly higher in epithelial cells than in ectopic stromal cells (R3 vs. R2) (Fig. 5), indicating that E-cadherin and Vimentin are specific markers for Ems epithelial cells. The above data demonstrate that ectopic endometrial epithelial cells have a tendency to transform into ectopic stromal cells when miR-218 is inhibited. In addition, E-cadherin and Vimentin expression was higher in eutopic endometrial epithelial cells than in normal eutopic endometrium (Fig. 4). These results were consistent with the data in ectopic endometrium of Ems patients (Fig. 4).

## Conclusions

Our findings reveal that miR-218-5p in endometrial microenvironment prevents the migration of ectopic endometrial stromal cells by inhibiting LASP1.

## Supplementary Information

**Additional file 1: sFig1.** Identification of endometrial stromal cells. **sTable 1.** The information for control group (Uterine Leiomyoma). **sTable 2.** The information for adenomyosis patient group.

## Data Availability

All data supported the conclusions during this study are included within the article.

## Abbreviations

LASP1: LIM and SH3 protein1
EdU: 5-Ethynyl-2’-deoxyuridine
Ems: Endometriosis
Ads: Adenomyosis
3’UTR: 3’ Untranslated region
EMT: Epithelial-mesenchymal transition
ESCs: Endometrial stromal cells
CEu: Control eutopic endometrium
AEu: Adenomyosis eutopic endometrium
AEc: Adenomyosis ectopic endometrium
EmL: Endometrial layer
MCL: Muscle layer
