# Supplementary material for: miR-218-5p in endometrial microenvironment prevents the migration of ectopic endometrial stromal cells by inhibiting LASP1
Source: Reprod Biol Endocrinol. 2022 Apr 4;20:64. doi: 10.1186/s12958-022-00928-z (PMC8978357; doi:10.1186/s12958-022-00928-z)
Supplement: Supplementary file 1 — Additional file 1: sFig1. Identification of endometrial stromal cells. sTable 1. The information for control group (Uterine Leiomyoma). sTable 2. The information for adenomyosis patient group. [file 12958_2022_928_MOESM1_ESM.docx]

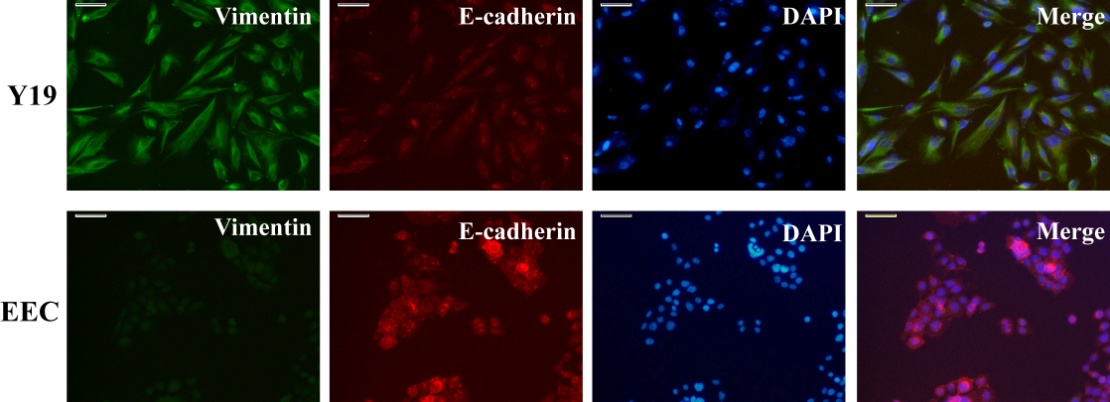


**sFig1. Identification of endometrial stromal cells.** Immunofluorescent analysis of Vimentin (Green) and E-cadherin (Red) in Y19 and EEC respectively. DAPI staining was included to visualize the cell nucleus (Blue), Scale bar=100 μm.

**sTable 1. The information for control group (Uterine Leiomyoma)**

| **No.** | **Age** | **Pathological diagnosis** |
| --- | --- | --- |
| **1** | 46 | Uterine Leiomyoma； |
| **2** | 39 | Uterine Leiomyoma； |
| **3** | 47 | Uterine Leiomyoma； |
| **4** | 48 | Uterine Leiomyoma； |
| **5** | 47 | Uterine Leiomyoma； |
| **6** | 39 | Uterine Leiomyoma； |
| **7** | 41 | Uterine Leiomyoma； |
| **8** | 41 | Uterine Leiomyoma； |
| **9** | 48 | Uterine Leiomyoma； |
| **10** | 48 | Uterine Leiomyoma； |
| **11** | 43 | Uterine Leiomyoma； |
| **12** | 48 | Uterine Leiomyoma； |
| **13** | 47 | Uterine Leiomyoma； |
| **14** | 49 | Uterine Leiomyoma； |
| **15** | 47 | Uterine Leiomyoma； |
| **16** | 43 | Uterine Leiomyoma； |
| **17** | 45 | Uterine Leiomyoma； |
|  |  |  |

**sTable 2. The information for adenomyosis patient group**

| **No.** | **Age** | **Pathological diagnosis** |
| --- | --- | --- |
| **1** | 43 | Adenomyoma |
| **2** | 49 | Adenomyoma |
| **3** | 49 | Adenomyosis |
| **4** | 41 | Adenomyosis |
| **5** | 42 | Adenomyosis |
| **6** | 45 | Adenomyosis |
| **7** | 50 | Adenomyoma |
| **8** | 43 | Adenomyoma |
| **9** | 41 | Adenomyosis |
| **10** | 47 | Adenomyosis |
| **11** | 51 | Adenomyosis |
| **12** | 48 | Adenomyosis |
| **13** | 39 | Adenomyosis |
| **14** | 43 | Adenomyoma |
| **15** | 41 | Adenomyosis |
| **16** | 44 | Adenomyosis |
